# Crystal structure of 6-eth­oxy­pyridin-1-ium-2-olate

**DOI:** 10.1107/S1600536814020224

**Published:** 2014-10-04

**Authors:** Kaijun Luo, Qing Guo, Yan Wang, Daibing Luo

**Affiliations:** a College of Chemistry and Materials Science, Sichuan Normal University, Chengdu, Sichuan 610068, People’s Republic of China; b Analytical and Testing Center, Sichuan University, Chengdu, Sichuan 610065, People’s Republic of China

**Keywords:** crystal structure, 6-eth­oxy­pyridin-1-ium-2-olate, zwitterion, hydrogen bonding, C—H⋯π inter­actions

## Abstract

In the title compound, C_7_H_9_NO_2_, all non-H atoms are essentially coplanar [r.m.s. deviation = 0.032 Å]. The largest deviation from the plane of the pyridine ring is 0.105 (6) Å for the terminal C atom of the eth­oxy group. In the crystal, mol­ecules are linked by pairs of N—H⋯O hydrogen bonds, forming inversion dimers. These dimers are further linked by C—H⋯π inter­actions and weak π–π inter­actions between pyridine rings [centroid–centroid distance = 4.023 (1) Å].

## Related literature   

For general background to 2-iodo-5-hy­droxy­pyridine derivatives and their applications, see: Cho *et al.* (2003 ▶); Hegmann *et al.* (2003 ▶); Savelon *et al.* (1998 ▶); Wang *et al.* (2012 ▶). For the synthesis of the title compound, see: Hutchinson *et al.* (2001 ▶); Seton *et al.* (2001 ▶).
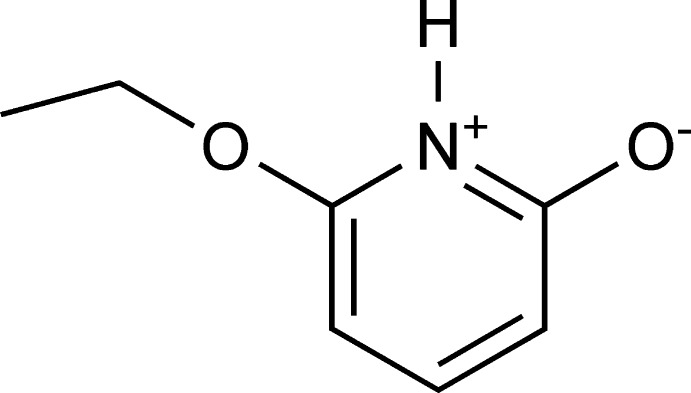


## Experimental   

### Crystal data  

C_7_H_9_NO_2_
*M* *_r_* = 139.15
Monoclinic, 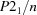

*a* = 8.3037 (11) Å
*b* = 7.0999 (6) Å
*c* = 12.0767 (15) Å
β = 93.402 (13)°
*V* = 710.74 (14) Å^3^
*Z* = 4
Mo *K*α radiation
μ = 0.10 mm^−1^
*T* = 293 K
0.3 × 0.3 × 0.2 mm

### Data collection  

Agilent Xcalibur Eos diffractometer
Absorption correction: multi-scan (*CrysAlis PRO*; Agilent, 2011 ▶) *T*_min_ = 0.606, *T*_max_ = 1.000
2957 measured reflections
1452 independent reflections
949 reflections with *I* > 2σ(*I*)
*R*_int_ = 0.018

### Refinement  

*R*[*F* ^2^ > 2σ(*F* ^2^)] = 0.055
*wR*(*F* ^2^) = 0.150
*S* = 1.09
1452 reflections
96 parameters
H atoms treated by a mixture of independent and constrained refinement
Δρ_max_ = 0.24 e Å^−3^
Δρ_min_ = −0.33 e Å^−3^

### 

Data collection: *CrysAlis PRO* (Agilent, 2011 ▶); cell refinement: *CrysAlis PRO*; data reduction: *CrysAlis PRO*; program(s) used to solve structure: *SUPERFLIP* (Palatinus & Chapuis, 2007 ▶); program(s) used to refine structure: *SHELXL97* (Sheldrick, 2008 ▶); molecular graphics: *OLEX2* (Dolomanov *et al.*, 2009 ▶); software used to prepare material for publication: *OLEX2*.

## Supplementary Material

Crystal structure: contains datablock(s) global, I. DOI: 10.1107/S1600536814020224/zq2227sup1.cif

Structure factors: contains datablock(s) I. DOI: 10.1107/S1600536814020224/zq2227Isup2.hkl

Supporting information file. DOI: 10.1107/S1600536814020224/zq2227Isup3.txt

Supporting information file. DOI: 10.1107/S1600536814020224/zq2227Isup4.cml

**Fig. 1.**
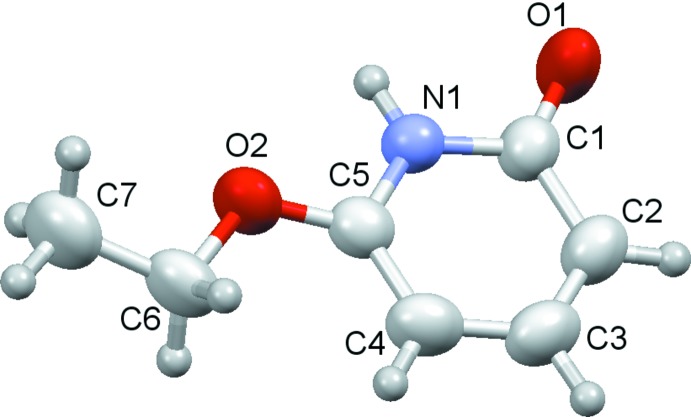
The molecular structure of the title complex, with non-hydrogen atoms labels and 50% probability displacement ellipsoids.

**Fig. 2.**
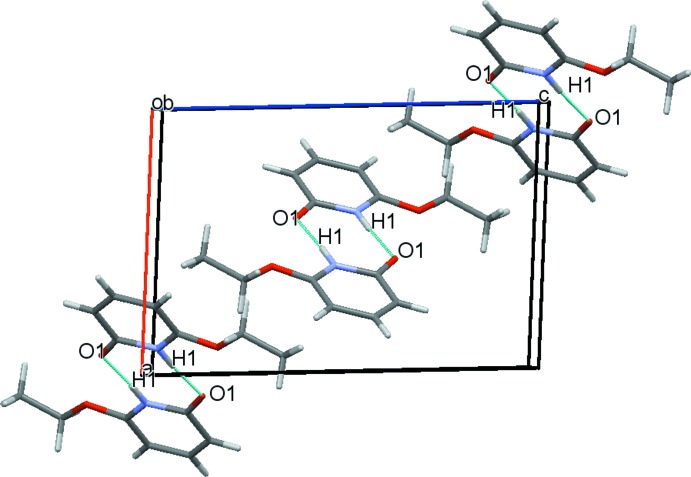
Packing of the title compound viewed along the b direction.

. DOI: 10.1107/S1600536814020224/zq2227fig1.tif
The mol­ecular structure of the title complex, with non-hydrogen atoms labels and 50% probability displacement ellipsoids.

b . DOI: 10.1107/S1600536814020224/zq2227fig2.tif
Packing of the title compound viewed along the *b* direction.

CCDC reference: 1023404

Additional supporting information:  crystallographic information; 3D view; checkCIF report

## Figures and Tables

**Table 1:** Hydrogen-bond geometry (, ) *Cg* is the centroid of the N1,C1C5 ring.

*D*H*A*	*D*H	H*A*	*D* *A*	*D*H*A*
N1H1O1^i^	0.87(2)	1.90(2)	2.762(2)	174(2)
C7H7*A* *Cg* ^ii^	0.96	2.90	3.792(3)	155

Symmetry codes: (i) 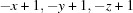; (ii) 
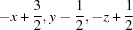
.

## supplementary crystallographic information

### S1. Comment

2-iodo-5-Hydroxypyridine derivatives are important materials for medicinal chemistry and material science. Recently, during the preparation of 2-iodo-5-hydroxypyridine, we accidentally got the 2-hydroxy-6-ethoxypyridine by-product, which is easy to sublimate to form the corresponding pyridinium by transferring hydrogen proton of 2-hydroxy to pyridine nitrogen atom. 2-hydroxy-6-ethoxypyridine can be obtained in a two-step synthesis from 2-iodo-5-nitropyridine.

In the title compound, C_7_H_9_NO_2_, all non-H atoms are essentially coplanar. The mean deviation for all non-hydrogen atoms of the molecule is 0.0315 Å, and the largest deviation from the least-squares plane of the six non-H atoms of the pyridine ring is 0.105 (6) Å for the terminal C atom of the ethoxy group. In the crystal, inversion-related molecules are linked through N1—H1···O1^i^ hydrogen bonds forming dimers [symmetry operators (i): -*x*+1, -*y*+1, -*z*+1]. These dimers are further linked by C—H···π interactions [H···centroid distance = 2.90 Å, C—H···centroid = 155°] between H7A and one pyridine ring [symmetry operator: -*x*+3/2, y-1/2, -*z+*1/2] and weak π–π interactions between pyridine rings [centroid–centroid distance = 4.023 (1) Å).

### S2. Experimental

A mixture of 2-iodo-5-nitropyridine (0.2 g, 0.8 mmol), SnCl_2_.2(H_2_O) (0.904 g, 4 mmol) and 25 ml ethanol was refluxed under inert atmosphere for 5 h. The mixture was evaporated to remove the solvent and the residue was extracted with ethyl acetate and H_2_O, and the organic layer was washed with 10% aqueous NaOH and H_2_O and dried over anhydrous MgSO_4_. The crude product was purified by flash chromatography [silica gel, petroleum ether: ethyl acetate (5:1)]. A light yellow solid (yield: 24%) of 2-ethoxy-5-aminopyridine was obtained. A solution of 2-ethoxy-5-aminopyridine (0.2 g, 0.90 mmol) and 40% fluoroboric acid (2 ml) was cooled down to 5 °C, then a 25% sodium nitrite solution (1.0 ml) was added. After stirring for 2 h at 5 °C, Cu_2_O (0.08 g, 0.54 mmol) and 30% hydrous CuNO_3_ (25 ml) were added to the solution and the mixture was stirred for 5 h at room temperature. The mixture was adjusted to pH 7 by 20% hydrous K_2_CO_3_ and filtered. The filtrate was extracted by ethyl acetate, the organic layer was washed with water and dried over MgSO_4_. The solvent was evaporated and the solid production was chromatographed on a silica gel column [petroleum ether: ethyl acetate (2:1). A white solid (yield: 18%), 2-hydroxy-6-ethoxypyridine, was obtained. Furthermore, single crystals of ¨the title compound were obtained by sublimation. ^1^H NMR (DMSO, 400 MHz) δ: 10.62 (s, 1 H), 748 (t, J = 8 Hz, 1 H), 6.12 (t, J = 8 Hz, 2 H), 4.18 (d, J = 8 Hz, 2 H), 1.26 (t, J = 8 Hz, 3 H).

### S3. Refinement

H atoms were refined as riding on their carriers with C—H = 0.93 Å and *U*_iso_(H) = 1.2U_eq_(C) for aromatic H atoms, with C—H = 0.97 Å and *U*_iso_(H) = 1.2U_eq_(C) for methylene H atoms, and with C—H = 0.96 Å and *U*_iso_(H) = 1.5U_eq_(C) for methyl H atoms, except H1 which was freely refined.

### Crystal data

**Table:**

C_7_H_9_NO_2_	*F*(000) = 296
*M**_r_* = 139.15	*D*_x_ = 1.300 Mg m^−^^3^
Monoclinic, *P*2_1_/*n*	Mo *K*α radiation, λ = 0.71073 Å
Hall symbol: -P 2yn	Cell parameters from 809 reflections
*a* = 8.3037 (11) Å	θ = 3.8–24.3°
*b* = 7.0999 (6) Å	µ = 0.10 mm^−^^1^
*c* = 12.0767 (15) Å	*T* = 293 K
β = 93.402 (13)°	Block, white
*V* = 710.74 (14) Å^3^	0.3 × 0.3 × 0.2 mm
*Z* = 4	

### Data collection

**Table:**

Agilent Xcalibur Eos diffractometer	1452 independent reflections
Radiation source: Enhance (Mo) X-ray Source	949 reflections with *I* > 2σ(*I*)
Graphite monochromator	*R*_int_ = 0.018
Detector resolution: 16.0874 pixels mm^-1^	θ_max_ = 26.4°, θ_min_ = 3.1°
ω scans	*h* = −9→10
Absorption correction: multi-scan (*CrysAlis PRO*; Agilent, 2011)	*k* = −5→8
*T*_min_ = 0.606, *T*_max_ = 1.000	*l* = −7→15
2957 measured reflections	

### Refinement

**Table:**

Refinement on *F*^2^	0 restraints
Least-squares matrix: full	H atoms treated by a mixture of independent and constrained refinement
*R*[*F*^2^ > 2σ(*F*^2^)] = 0.055	*w* = 1/[σ^2^(*F*_o_^2^) + (0.0675*P*)^2^ + 0.0301*P*] where *P* = (*F*_o_^2^ + 2*F*_c_^2^)/3
*wR*(*F*^2^) = 0.150	(Δ/σ)_max_ = 0.001
*S* = 1.09	Δρ_max_ = 0.24 e Å^−^^3^
1452 reflections	Δρ_min_ = −0.33 e Å^−^^3^
96 parameters	

### Special details

**Table:**

Geometry. All e.s.d.'s (except the e.s.d. in the dihedral angle between two l.s. planes) are estimated using the full covariance matrix. The cell e.s.d.'s are taken into account individually in the estimation of e.s.d.'s in distances, angles and torsion angles; correlations between e.s.d.'s in cell parameters are only used when they are defined by crystal symmetry. An approximate (isotropic) treatment of cell e.s.d.'s is used for estimating e.s.d.'s involving l.s. planes.
Refinement. Reflections were merged by *SHELXL* according to the crystal class for the calculation of statistics and refinement.

### Fractional atomic coordinates and isotropic or equivalent isotropic displacement parameters (Å^2^)

**Table:**

	*x*	*y*	*z*	*U*_iso_*/*U*_eq_	
C1	0.6392 (2)	0.3124 (3)	0.59009 (17)	0.0554 (5)	
C2	0.7456 (3)	0.1850 (3)	0.64769 (19)	0.0642 (6)	
H2	0.7774	0.2064	0.7218	0.077*	
C3	0.8004 (3)	0.0333 (3)	0.5951 (2)	0.0688 (7)	
H3	0.8694	−0.0493	0.6344	0.083*	
C4	0.7581 (3)	−0.0052 (3)	0.4841 (2)	0.0663 (7)	
H4	0.7979	−0.1108	0.4492	0.080*	
C5	0.6563 (2)	0.1176 (2)	0.42860 (18)	0.0521 (5)	
C6	0.6502 (3)	−0.0472 (3)	0.2572 (2)	0.0683 (7)	
H6A	0.6240	−0.1649	0.2928	0.082*	
H6B	0.7658	−0.0431	0.2495	0.082*	
C7	0.5627 (3)	−0.0328 (4)	0.1469 (2)	0.0824 (8)	
H7A	0.5910	−0.1376	0.1017	0.124*	
H7B	0.5919	0.0825	0.1117	0.124*	
H7C	0.4486	−0.0338	0.1557	0.124*	
N1	0.60093 (19)	0.2701 (2)	0.48106 (14)	0.0507 (5)	
H1	0.538 (3)	0.350 (3)	0.445 (2)	0.082 (8)*	
O1	0.58033 (19)	0.4567 (2)	0.63138 (12)	0.0762 (5)	
O2	0.60054 (17)	0.11012 (17)	0.32261 (12)	0.0629 (5)	

### Atomic displacement parameters (Å^2^)

**Table:**

	*U*^11^	*U*^22^	*U*^33^	*U*^12^	*U*^13^	*U*^23^
C1	0.0558 (12)	0.0567 (11)	0.0530 (12)	−0.0021 (9)	−0.0032 (10)	0.0008 (10)
C2	0.0651 (14)	0.0644 (12)	0.0619 (14)	0.0015 (10)	−0.0066 (11)	0.0103 (11)
C3	0.0624 (14)	0.0641 (13)	0.0792 (17)	0.0075 (11)	−0.0027 (12)	0.0185 (12)
C4	0.0646 (14)	0.0521 (11)	0.0828 (18)	0.0098 (10)	0.0096 (13)	0.0026 (11)
C5	0.0504 (11)	0.0462 (10)	0.0603 (13)	−0.0039 (8)	0.0081 (10)	−0.0007 (9)
C6	0.0732 (15)	0.0593 (12)	0.0738 (16)	0.0059 (10)	0.0173 (13)	−0.0164 (11)
C7	0.0939 (19)	0.0759 (15)	0.0782 (18)	0.0062 (13)	0.0121 (15)	−0.0260 (13)
N1	0.0508 (10)	0.0472 (9)	0.0536 (10)	0.0040 (7)	0.0000 (8)	−0.0025 (8)
O1	0.0997 (13)	0.0716 (10)	0.0550 (10)	0.0237 (9)	−0.0148 (9)	−0.0131 (7)
O2	0.0732 (10)	0.0531 (8)	0.0628 (10)	0.0086 (6)	0.0059 (8)	−0.0118 (7)

### Geometric parameters (Å, º)

**Table:**

C1—C2	1.418 (3)	C5—O2	1.336 (2)
C1—N1	1.369 (2)	C6—H6A	0.9700
C1—O1	1.251 (2)	C6—H6B	0.9700
C2—H2	0.9300	C6—C7	1.483 (3)
C2—C3	1.344 (3)	C6—O2	1.442 (2)
C3—H3	0.9300	C7—H7A	0.9600
C3—C4	1.392 (3)	C7—H7B	0.9600
C4—H4	0.9300	C7—H7C	0.9600
C4—C5	1.363 (3)	N1—H1	0.87 (3)
C5—N1	1.349 (2)		
			
N1—C1—C2	115.75 (19)	C7—C6—H6A	110.2
O1—C1—C2	125.0 (2)	C7—C6—H6B	110.2
O1—C1—N1	119.25 (17)	O2—C6—H6A	110.2
C1—C2—H2	120.1	O2—C6—H6B	110.2
C3—C2—C1	119.9 (2)	O2—C6—C7	107.33 (18)
C3—C2—H2	120.1	C6—C7—H7A	109.5
C2—C3—H3	118.7	C6—C7—H7B	109.5
C2—C3—C4	122.6 (2)	C6—C7—H7C	109.5
C4—C3—H3	118.7	H7A—C7—H7B	109.5
C3—C4—H4	121.3	H7A—C7—H7C	109.5
C5—C4—C3	117.5 (2)	H7B—C7—H7C	109.5
C5—C4—H4	121.3	C1—N1—H1	116.0 (17)
N1—C5—C4	120.1 (2)	C5—N1—C1	124.13 (18)
O2—C5—C4	127.98 (19)	C5—N1—H1	119.9 (17)
O2—C5—N1	111.91 (17)	C5—O2—C6	117.46 (16)
H6A—C6—H6B	108.5		
			
C1—C2—C3—C4	0.5 (3)	C7—C6—O2—C5	175.32 (18)
C2—C1—N1—C5	0.8 (3)	N1—C1—C2—C3	−0.7 (3)
C2—C3—C4—C5	−0.3 (3)	N1—C5—O2—C6	−179.67 (16)
C3—C4—C5—N1	0.3 (3)	O1—C1—C2—C3	179.4 (2)
C3—C4—C5—O2	179.6 (2)	O1—C1—N1—C5	−179.39 (18)
C4—C5—N1—C1	−0.6 (3)	O2—C5—N1—C1	−179.95 (16)
C4—C5—O2—C6	1.0 (3)		

### Hydrogen-bond geometry (Å, º)

Cg is the centroid of the N1,C1–C5 ring.

**Table:**

*D*—H···*A*	*D*—H	H···*A*	*D*···*A*	*D*—H···*A*
N1—H1···O1^i^	0.87 (2)	1.90 (2)	2.762 (2)	174 (2)
C7—H7*A*···*Cg*^ii^	0.96	2.90	3.792 (3)	155

Symmetry codes: (i) −*x*+1, −*y*+1, −*z*+1; (ii) −*x*+3/2, *y*−1/2, −*z*+1/2.
