# Supplementary figures and images for: Crystal structure of 6-eth­oxy­pyridin-1-ium-2-olate
Source: Acta Crystallogr Sect E Struct Rep Online. 2014 Oct 4;70(Pt 11):o1146. doi: 10.1107/S1600536814020224 (PMC4257288; doi:10.1107/S1600536814020224)

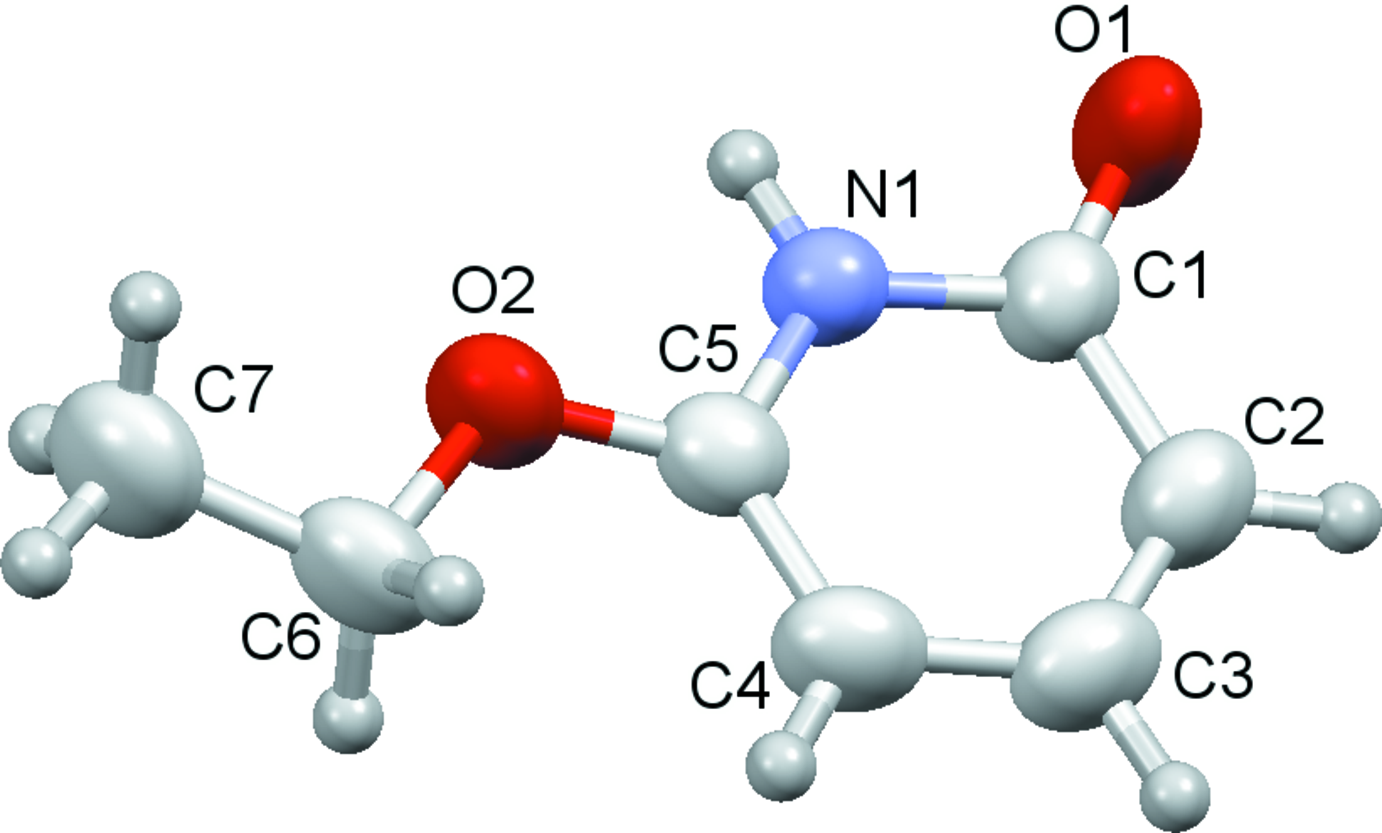

Supplement: Supplementary file 5 [file e-70-o1146-fig1.tif]

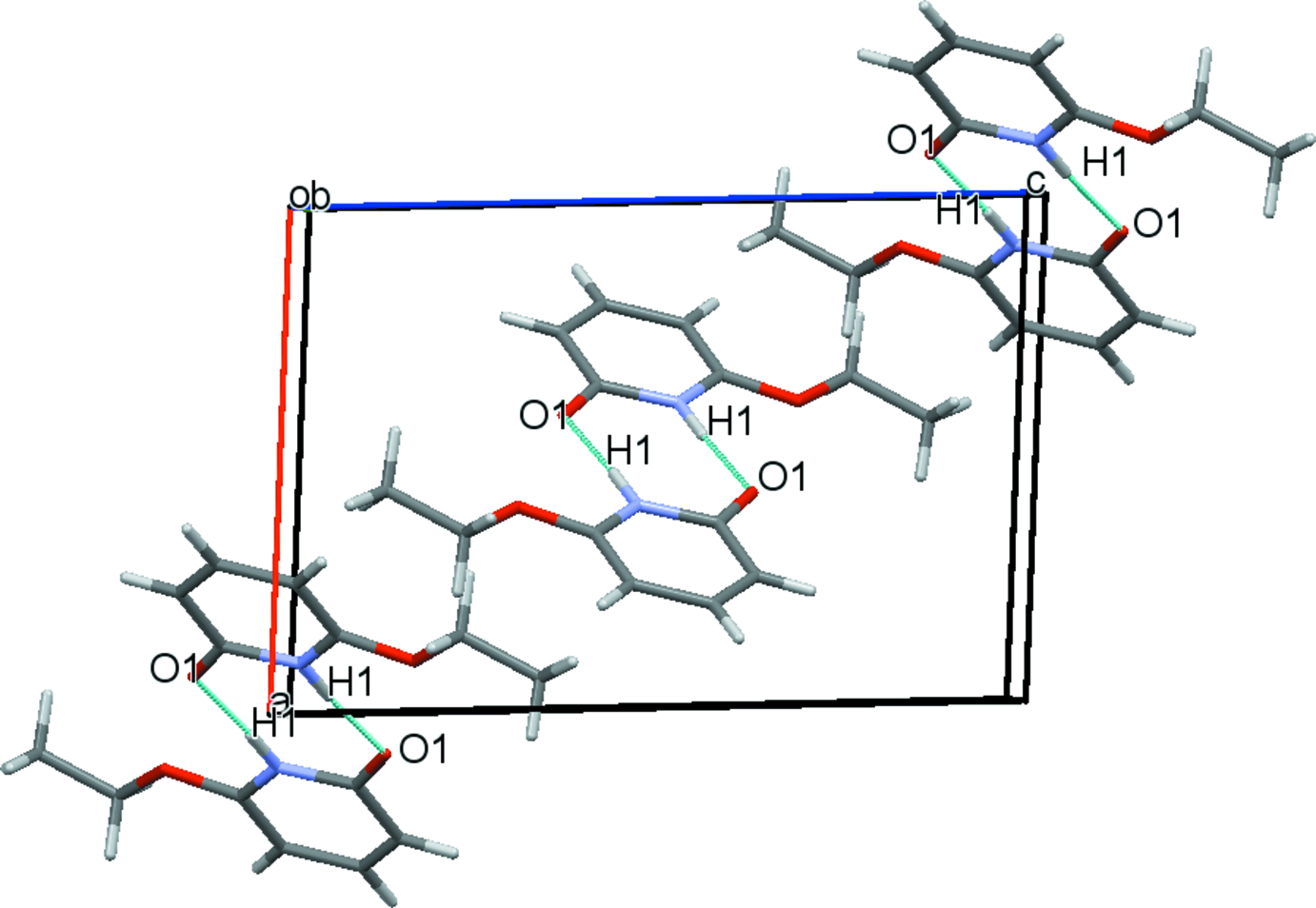

Supplement: Supplementary file 6 [file e-70-o1146-fig2.tif]
